# The chromatin remodeller CHD8 is required for E2F-dependent transcription activation of S-phase genes

**DOI:** 10.1093/nar/gkt1161

**Published:** 2013-11-20

**Authors:** Alicia Subtil-Rodríguez, Elena Vázquez-Chávez, María Ceballos-Chávez, Manuel Rodríguez-Paredes, José I. Martín-Subero, Manel Esteller, José C. Reyes

**Affiliations:** ^1^Molecular Biology Department, Centro Andaluz de Biología Molecular y Medicina Regenerativa (CABIMER), Consejo Superior de Investigaciones Científicas (CSIC), Av. Americo Vespucio 41092 Seville, Spain, ^2^Cancer Epigenetics and Biology Program, Bellvitge Biomedical Research Institute, L’Hospitalet, Barcelona, Spain and ^3^Department of Anatomic Pathology, Pharmacology and Microbiology, University of Barcelona, Spain

## Abstract

The precise regulation of S-phase–specific genes is critical for cell proliferation. How the repressive chromatin configuration mediated by the retinoblastoma protein and repressor E2F factors changes at the G1/S transition to allow transcription activation is unclear. Here we show ChIP-on-chip studies that reveal that the chromatin remodeller CHD8 binds ∼2000 transcriptionally active promoters. The spectrum of CHD8 target genes was enriched in E2F-dependent genes. We found that CHD8 binds E2F-dependent promoters at the G1/S transition but not in quiescent cells. Consistently, CHD8 was required for G1/S-specific expression of these genes and for cell cycle re-entry on serum stimulation of quiescent cells. We also show that CHD8 interacts with E2F1 and, importantly, loading of E2F1 and E2F3, but not E2F4, onto S-specific promoters, requires CHD8. However, CHD8 recruiting is independent of these factors. Recruiting of MLL histone methyltransferase complexes to S-specific promoters was also severely impaired in the absence of CHD8. Furthermore, depletion of CHD8 abolished E2F1 overexpression-dependent S-phase stimulation of serum-starved cells, highlighting the essential role of CHD8 in E2F-dependent transcription activation.

## INTRODUCTION

The decision about when DNA should be replicated is probably one of the most controlled processes in the cell. Therefore, repression of genes required for proliferation during G0 and early G1 phases of the cell cycle, and activation of genes specific for S phase, are tightly controlled processes that are critical during normal differentiation and tissue homeostasis and that seem to be disregulated in most cancers (1,2). E2Fs comprise a large family of transcription factors that bind promoter regions and are of paramount importance in regulating cell proliferation (3,4). Based on functional studies and amino acid sequence analyses, classical E2F family members can be divided into the two canonical classes: repressor E2Fs (E2F4 and E2F5) or activator E2Fs (E2F1–E2F3), although exceptions to this canonical classification has been reported (5). During interphase or under quiescent conditions, E2F4 and E2F5 associate with the retinoblastoma (RB) family of pocket proteins (RB, p107 and p130) and repressor complexes to inhibit transcription of G1/S transition genes [reviewed in (6)]. On growth factor stimulation, specific cyclin-dependent kinases phosphorylate pocket proteins and disrupt their inhibitory activity. This leads to the induction of activator E2Fs, which then substitute their repressor counterparts and promote the expression of genes required for S phase, through a unclear mechanism that involve histone modifications and chromatin remodelling [reviewed in (7)].

CHD8 is a human ATP-dependent chromatin remodelling protein of the SNF2 family, homologue of the Trithorax group (TrxG) protein Kismet from *Drosophila* (8,9). Ectopic expression studies have shown that CHD8 represses β-catenin target genes, and suppresses p53-dependent activation and apoptosis, by promoting histone H1 recruitment (10–13). However, biochemical studies have shown that CHD8 copurifies with transcription activation complexes, such as the MLL complexes (11,14–16), and with elongating RNAPII (17). Accordingly, the activity of CHD8 as a transcription activator has also been reported. CHD8 cooperates with the androgen receptor for gene activation (18) and activates expression of cyclin E2 (*CCNE2*) and thymidylate synthetase (*TYMS*) genes (17,19).

Here we show by ChIP-on-chip (ChIP-chip) analysis that CHD8 binds to the promoter of ∼2000 active genes that are also enriched for H3K4me2 and H3K4me3 marks. In agreement with this result, artificial recruitment of CHD8 to a synthetic promoter activated transcription. We found that E2F binding motives were strongly enriched in promoters containing CHD8, suggesting that CHD8 may be involved in E2F-dependent transcription. Analysis of a subset of E2F and CHD8 targets demonstrated that CHD8 is crucial for the timely activation of E2F-dependent promoters. Consistently, CHD8 was essential for normal cell cycle re-entry on serum stimulation. The presence of CHD8 is required for E2F1, E2F3 and the MLL histone methyltransferase complexes to be loaded onto G1/S transition promoters. Therefore, our data demonstrate that CHD8 is a critical factor for gene activation during the G1/S transition.

## MATERIALS AND METHODS

### Cell culture and experimental conditions

C33A (human cervix carcinoma), HEK-293 (human embryonic kidney) and COS7 (green monkey kidney fibroblast) cell lines were maintained in Dulbecco's modified Eagle's medium (DMEM), and the RPE-1 (immortalized retina epithelium) cell line was maintained in DMEM F12 Ham. In both cases, medium was supplemented with 7% fetal bovine serum (FBS), 100 U/ml penicillin and 100 μg/ml streptomycin, and cells were cultivated in a 37°C incubator with 5% CO2. For RPE-1 cells synchronization, cells were grown exponentially (in 10% FBS) and then subjected to serum starvation during 48 h. After that, cells were collected (0%) or medium was replaced with fresh medium supplemented with 20% FBS for the indicated times. For cell cycle analysis, cells were washed with cold 1 × PBS, fixed in 70% ethanol and stained with an analysis solution of 0.25 mg/ml ribonuclease A (Sigma) and 0.05 mg/ml propidium iodide (Sigma) in 1 × PBS. Samples were analysed using a FACS Calibur machine (BD Biosciences), CellQuest analysis software and ModFit program.

### ChIP assays

ChIP assays were performed as described (20) using anti-CHD8 (A301-224 A) and anti-E2F1 (A300-766 A) from Bethyl Laboratories; anti-RNAPII (N-20) (sc-899), anti-E2F3 (sc-878) and anti-E2F4 (sc-1082) from Santa Cruz Biotechnology; anti-WDR5 (ab56919), anti-H3K4me3 (ab8580) and anti-H3K4me2 (ab32356) from Abcam and an anti-CHD8 antibody home made (17). Chromatin was sonicated to an average fragment size of 400–500 bp using the Diagenode Bioruptor. Rabbit IgG (Sigma) was used as a control for non-specific interactions. Input was prepared with 10% of the chromatin material used for immunoprecipation. Input material was diluted 1:10 before PCR amplification. Quantification of immunoprecipitated DNA was performed by real-time PCR (qPCR) with the Applied Biosystems 7500 FAST real-time PCR system, using Applied Biosystems Power SYBR green master mix. Sample quantifications by qPCR were performed in triplicate. Sequences of all oligonucleotides are provided in Supplementary Table S1. Data are the average of at least three independent experiments.

### ChIP-chip microarray hybridization and analysis

ChIP was performed as described earlier in text using the following antibodies: anti-CHD8 (A301-224 A, Bethyl Laboratories), anti-H3K4me3 (ab8580, Abcam) and anti-H3K4me2 (ab32356, Abcam). Then, input (100 ng) and ChIP DNA were amplified with the GenomePlex complete whole genome amplification WGA2 kit (Sigma), according to the manufacturer’s recommended protocol, and subsequently purified with Qiaquick Qiagen columns. Input and ChIP-amplified DNA were labelled with Alexa Fluor 5 or Alexa Fluor 3 propargyl–linked fluorophores with the BioPrime total FFPE genomic labelling system following the manufacturer's instructions (Invitrogen). Labelled samples were purified with silica-based PureLink spin columns (Invitrogen). Labelled amplified DNAs were then combined and hybridized to an Agilent's 1 Mb Custom Human Promoter-CpG island microarray, designed with Agilent's eArray application (https://earray.chem.agilent.com/earray). The custom microarray was made out of two different microarrays for epigenetic studies designed by Agilent: (i) human promoter ChIP-on-chip set (2 × 244 k), which contained extended promoter regions ranging from 5.5 kb upstream and 2.5 kb downstream of the TSS, of ∼17 000 promoters; and (ii) human DNA methylation microarray (1 × 244 k), which contained 27 627 expanded CpG islands and 5081 unmethylated regions. Hybridization and washes were performed as described by Agilent in a SureHyb hybridization chamber (Agilent). Arrays were then immediately scanned on a G2565C DNA microarray scanner (Agilent). Images were quantified using Agilent Feature Extraction Software (version 10.7). Raw ratios between immunoprecipitated and input DNAs in log2 scale were analysed with the R software (www.r-project.org) using the Ringo package adapted for Agilent arrays (21) available through Bioconductor. Data were normalized and smoothed, and peaks were detected using the upperBoundNull non-parametric approach. A significantly enriched peak was defined by the following criteria: the minimum number of enriched probes within a peak was set to three, the maximum amount of base pairs at which enriched probes were condensed into one enriched peak was set to 600 and the *P *was required to be <0.01. Data have been deposited in GEO with accession number GSE51564.

### Microarray expression analysis

Total RNA was isolated in triplicate from exponentially growing C33A cells using RNeasy Mini Kit (Qiagen). Purity and quality of isolated RNA were assessed by RNA 6000 Nano assay on a 2100 Bioanalyzer (Agilent Technologies, Santa 6 Clara, CA, USA). RNA (100 ng) was used for production of end-labelled biotinylated ssDNA. Labelled ssDNA was hybridized to the GeneChip® human Gene 1.0 ST array oligonucleotide microarray (Affymetrix, Santa Clara, CA, USA) according to manufacturer’s recommendations. The arrays were scanned using the GeneChip Scanner 3000 7 G (Affymetrix), and raw data were extracted from the scanned images and analysed with the Affymetrix GeneChip Command Console Software (Affymetrix). The raw array data were preprocessed and normalized using the Robust Multichip Average method (22). Data were further processed using oneChannelGUI (23). The log2 intensities for each probe were used for further analysis. Data have been deposited in GEO with accession number GSE48926.

### Luciferase reporter assays

HEK-293 cells were transfected by calcium phosphate with the indicated plasmids following the protocol described in (24). Vectors encoding the luciferase reporter gene under the control of adenovirus early gene IV (EIV) minimal promoter, with or without 5 × Gal4 DNA binding sites (p5 × Gal4-EIV-LUC and pEIVluc), were a gift from C. Muchardt. pSG5-Gal4-HP1α, encoding the HP1α protein fused to the Gal4 DNA-binding domain, was a gift from R. Losson. pSG5-Gal4-CHD8 or pSG5-Gal4-CHD8-K842A was generated by standard PCR and cloning techniques (cloning strategies details will be provided on request). All transfections were normalized by measuring the β-galactosidase activity of the samples, using cotransfection with pAdRSV-βgal vector, a gift from P. Charnay (25).

### RNAi experiments and transfections

All siRNAs were transfected using Oligofectamine (Invitrogen) according to the manufacturer’s instructions. The following siRNAs were used: siCHD8#1, 5′-GAGCAAGCUCAACACCAUC-3′; siCHD8#2, 5′-GUGCUUCUGGAAUGUUAAC-3′; siCHD8#3 5′-GAACUACUCCUAUCUGCAU-3′; for siE2F1, 5′-AAGUCACGCUAUGAGACCUCA-3′; for siE2F3, 5′-CGUCCAAUGGAUGGGCUGC-3′; and siCt, 5′-CGUACGCGGAAUACUUCGA-3′. After transfection, cells were grown in exponential conditions or, as indicated, the medium was replaced by fresh medium without serum. After 48 h in serum-free conditions, cells were collected (0%) or medium was replaced with fresh medium supplemented with 20% FBS for the indicated times. The downregulation of CHD8 (Figures 2B, 3C and 4B), E2F1 and E2F3 (Figure 3E) expression was determined by western blotting. Anti-α-tubulin antibody (Clone DM1A) from Sigma Aldrich was used as a loading control. When indicated, 24 h after siRNA transfection, cells were transfected with plasmids expressing E2F1 (pCMV-E2F1, provided by C. Muchardt), E1A (pCMV-12 S, provided by F. Thierry) or empty vectors as a control, using Lipofectamine (Invitrogen) according to the manufacturer’s instructions. After 24 h in serum-free conditions, cells were collected for cell cycle analysis or expression analysis by RT-qPCR.

### RNA extraction and RT-qPCR

Total RNA was prepared by using the RNeasy Kit (Qiagen), as described in the manufacturer’s instructions; the step of DNase I digestion was included to avoid potential DNA contamination. cDNA was generated from 800 ng of total RNA using Superscript First Strand Synthesis System (Invitrogen). cDNA (2 μl) was used as a template for RT-qPCR. Gene products were quantified by qPCR with the Applied Biosystems 7500 FAST real-time PCR system, using Applied Biosystems Power SYBR green master mix. Sequences of all oligonucleotides are provided in Supplementary Table S1. Values were normalized to the expression of the *ACTB* housekeeping gene. Each experiment was performed at least in duplicate, and qPCR quantifications were performed in triplicate.

### Immunofluorescence

A cilium assembly/disassembly assay was performed as previously described (26). Specifically, cells were starved in serum-free medium for 48 h to induce cilium formation. Serum was then added back to the medium to stimulate cilium resorption and cell cycle re-entry. Cells were harvested at various time points (0 h, 2 h and 24 h) and fixed with 100% methanol for 7 min, permeabilized for 5 min in 0.5% Triton X-100/PBS, washed and then blocked in 3% BSA/PBS. Monoclonal acetylated α-tubulin mouse antibody (clone 611B) and monoclonal γ-tubulin mouse antibody (clone GTU-88) were purchased from Sigma Aldrich. Secondary antibodies used were FITC-conjugated goat anti-rabbit IgG and Texas Red goat anti-mouse IgG (Jackson ImmunoResearch). Cells were examined under a motorized upright wide-field microscope (DM6000B; Leica). Image analysis was carried out using Leica and Adobe Photoshop software.

### Coimmunoprecipitation assays

Whole-cell extracts from cells were obtained by lysing the cells in immunoprecipitation (IP) buffer [50 mM Tris–HCl (pH 8), 150 mM NaCl, 1 mM EDTA (pH 8), 1% Triton X-100, 1 mM PMSF and protease inhibitors cocktail from GE Healthcare]. The extracts were pre-cleared for 3 h at 4°C with protein A or protein G–sepharose beads (GE Healthcare) pre-equilibrated in the same buffer. The pre-cleared extracts were then incubated overnight at 4°C with 2 μg of the appropriate antibody. Rabbit or mouse purified IgG (Sigma) were used as a control. Immunocomplexes were purified by adding 30 μl of 50% protein A or protein G sepharose beads. Finally, after three washes with IP buffer, bound proteins were eluted by boiling the beads in Laemmli sample buffer containing 5% β-mercaptoethanol, separated by SDS/PAGE and visualized by western blot with the indicated antibodies using ECL Plus (GE Healthcare), according to the manufacturer’s instructions.

## RESULTS

### CHD8 binds a subset of transcriptionally active genes.

**Figure 1.**
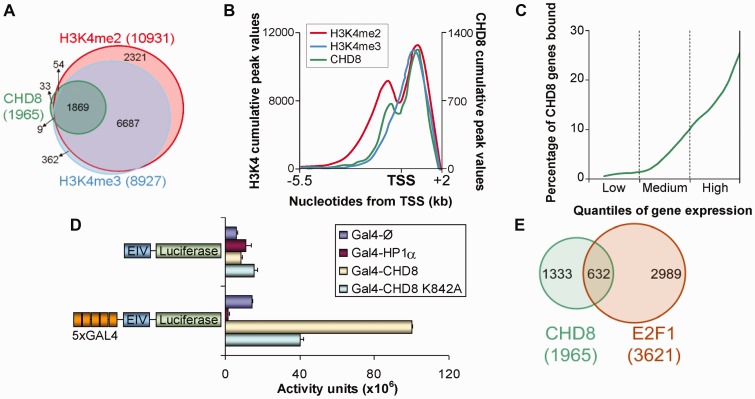
CHD8 binds a subset of transcriptionally active promoters. (**A**) Venn-diagram showing overlap between CHD8 targets (green) and H3K4me2- (red) or H3K4me3- (blue) enriched genes. (**B**) Distribution of CHD8 (green), H3K4me2 (red) and H3K4me3 (blue) enrichment regions relative to the TSS of RefSeq genes. (**C**) All genes were binned into ten quantiles based on their transcribed level, as determined by Affymetrix microarrays [Gene Expression Omnibus (GEO) Accession GSE48926] (*x*-axis). The *y*-axis shows the average CHD8 binding frequency for all genes in each quantile. (**D**) Luciferase reporter plasmids harbouring an EIV minimal promoter, and containing or not upstream 5×Gal4 DNA binding sites, were cotransfected with empty pSG5 vector (Gal4-Ø) or pSG5-Gal4-HP1α (Gal4-HP1α) as a negative control or with pSG5-Gal4-CHD8 (Gal4-CHD8) or pSG5-Gal4-CHD8-K842A (Gal4-CHD8 K842A) (which encode for the given proteins fused to the Gal4 DNA-binding domain), in 293 cells. Data are expressed as the mean activity from three independent experiments ± SD values. (**E**) Venn-diagram showing overlap between CHD8 (green) and E2F1 (brown) binding genes. E2F1 data set in HeLa-S3 cells (GSM935484) was obtained from ENCODE database stored in GEO. A *P-*value of 3.3 × 10^−57^ was calculated using the hypergeometric distribution.

To investigate the target genes of CHD8, ChIP-chip analysis of C33A human cervical carcinoma cells was performed using a custom-designed promoters and CpG island Agilent microarray. Two independent ChIP-chip experiments produced similar enrichment ratios (Pearson coefficient, *r* = 0.664) (Supplementary Figure S1A). CHD8 was associated (*P* < 0.01) with the promoter of 2887 and 2476 genes in each experiment, respectively. The 1965 promoter regions that were enriched for CHD8 in both experiments, were thus considered to be CHD8 target genes (Supplementary Table S2). CHD8 occupancy was confirmed by ChIP-qPCR in six selected target genes (*BRD2*, *RPS18*, *HMG20A*, *CAPZA1*, *COPZ1 *and *N4BP1*), in contrast, CHD8 was not found in three non-target genes (*ABCA10*, *DEFB133*, *MSA4A13*), validating the ChIP-chip results (Supplementary Figure S1B). We also confirmed that CHD8 was absent from the 3′ part of the *CCNE2* gene (Supplementary Figure S1C). We have previously shown that CHD8 binds H3K4me2 and, to a lesser extent, H3K4me3, through its tandem chromo-domains (17). To investigate whether CHD8 binds specifically to the promoters containing these marks, we also determined the distribution of H3K4me2 and H3K4me3 by ChIP-chip in C33A cells (Supplementary Table S3). About 95% (1869 promoters) of the CHD8 target promoters also contained H3K4me2 and H3K4me3 marks (Figure 1A). The CHD8-enriched probe distribution relative to the transcription start site (TSS) strongly overlapped with that of the H3K4me2 and H3K4me3 signals (Figure 1B). Thus, as methylated H3K4, CHD8 was enriched not only at the promoters but also at the 5′-end of gene bodies. Furthermore, a strong correlation between CHD8 enrichment intensities and those of H3K4me3 (Pearson *r* = 0.643) and H3K4me2 (Pearson r = 0.659) was found (Supplementary Figure S1A). As H3K4me3 and H3K4me2 modifications are normally associated with active promoters these data suggested that CHD8 is enriched in transcriptionally active genes. In agreement with this, most of the CHD8 target genes presented a medium or high transcriptional activity, based on C33A cells transcripts levels determined from Affymetrix microarrays (Figure 1C).

To investigate the functional effect of CHD8 binding to a promoter, we have evaluated the consequences of artificially recruiting a Gal4-CHD8 fusion protein to a minimal EIV adenovirus promoter with five Gal4 DNA-binding sites that control the expression of a luciferase reporter gene. As control, we also determined the effect of recruiting the typical repressor factor heterochromatin protein 1 alpha (HP1α) (27). As shown in Figure 1D, Gal4-HP1α expression led to a 10-fold decreased reporter gene expression. In contrast, expression of Gal4-CHD8 increased ∼7-fold luciferase activity indicating that CHD8 is able to activate transcription when artificially recruited to a promoter. Interestingly, a point mutation in the ATP binding site of CHD8 (CHD8-K842A), which abolished the *in vitro* chromatin remodelling activity of the protein (11), partially impaired Gal4-CHD8-dependent transcription activation. Expression of the reporter system lacking Gal4 binding sites was largely independent of the presence of Gal4 fusion proteins (Figure 1D). Therefore, our data indicate that CHD8 is able to activate transcription when recruited to a promoter, and that this function is, at least in part, dependent on its chromatin remodelling activity.

Gene Ontology (GO) analyses using DAVID (28) software indicated that a large proportion of the CHD8 target genes identified by ChIP-chip are involved in macromolecular biosynthetic processes (transcription, mRNA processing and translation), chromatin organization (histones and chromatin-associated factors) and cell cycle (Supplementary Figure S1D). Consistently, we found that CHD8 targets were strongly enriched in E2F binding sites (*P* = 1.31 × 10^−^^64^) (Supplementary Figure S1E). Thus, 67% of the CHD8-bound promoters also contain E2F binding sites (1314 promoters). Furthermore, using ENCODE ChIP-seq data, we found that 32% of CHD8 target promoters were occupied by E2F1 in HeLa cells (*P* = 3.3 × 10^−^^57^), another cervical carcinoma cell line (Figure 1E). These data suggested that CHD8 has a role in regulating E2F target genes.

### CHD8 controls expression of E2F targets.

**Figure 2.**
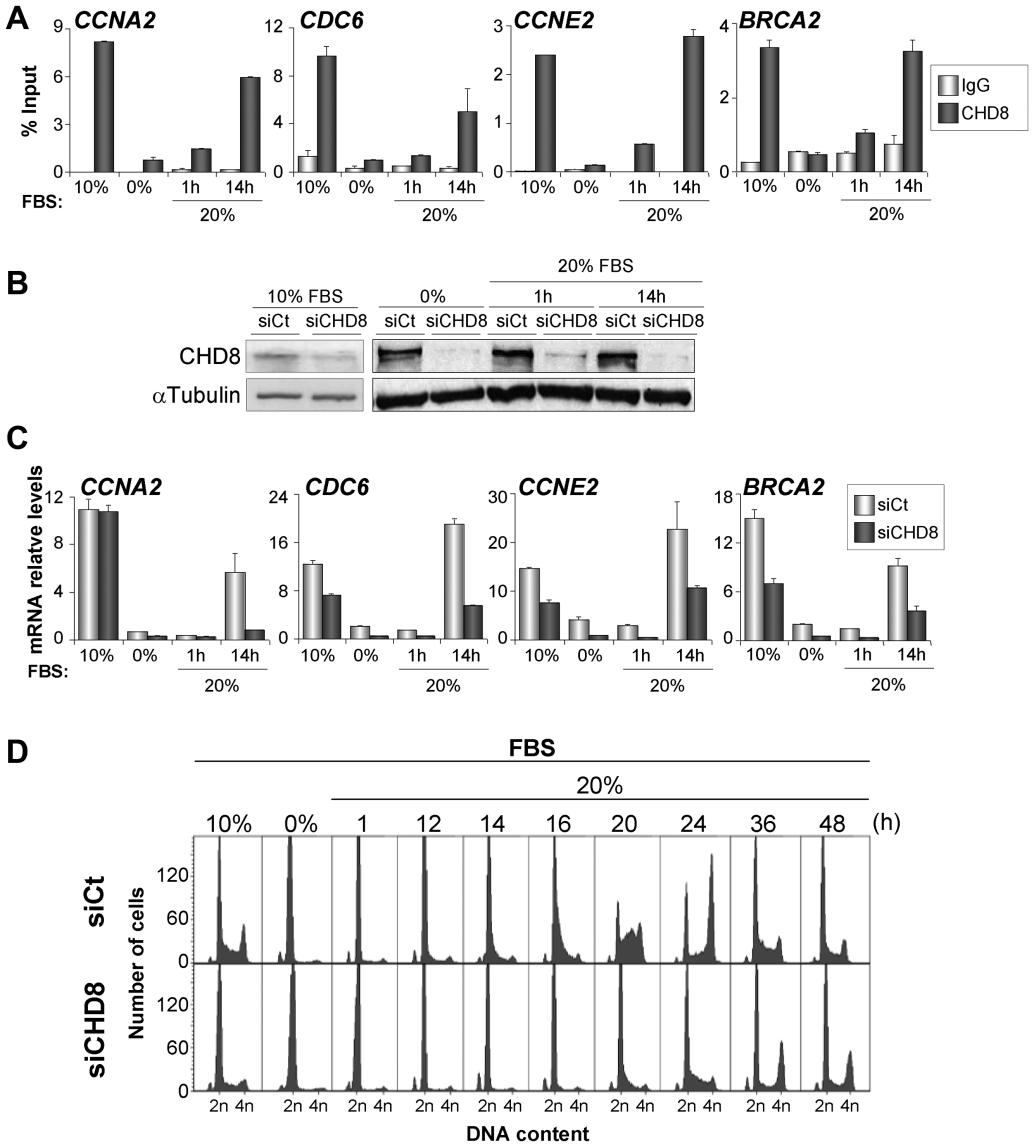
CHD8 binds and controls expression of E2F-dependent genes. (**A**) ChIP analysis of CHD8 on selected E2F-dependent genes. RPE1 cells were either grown exponentially (in 10% FBS) or serum-starved for 48 h (0%) and then serum stimulated (FBS 20%) for the indicated times. (**B**) Efficiency of CHD8 knockdown was analysed by western blotting of proliferating (10% FBS) or serum starved (0% FBS) and then serum stimulated (1 h and 10 h 20% FBS) RPE1 cells that had been transfected with control siRNA (siCt) or siRNA against CHD8 (siCHD8) using siCHD8#1. (**C**) RT-qPCR analysis of expression of selected E2F-dependent genes in RPE1 cells transfected with control siRNA (siCt) or siRNA against CHD8 (siCHD8). Cells were serum starved and then serum stimulated as in (A). (**D**) Cell cycle analysis by flow cytometry of control (siCt) or CHD8-depleted (siCHD8) RPE1 cells, either grown exponentially (10%) or serum-starved and then stimulated with 20% FBS for the indicated times. A representative experiment is shown. (A and C) Data (% input or mRNA relative level) are the mean of at least *n* = 6 qPCR reactions from three independent experiments. Error bars represent ± SD values.

We next investigated how CHD8 affects the cell cycle-dependent expression of a subset of E2F target genes in a non-tumorigenic cell line. For this, we used the *hTERT*-immortalized retinal pigment epithelial cell line, RPE1, which presents a normal karyotype and can be efficiently synchronized by serum starvation (29). ChIP-qPCR analysis in asynchronously growing RPE1 cells, using two different anti-CHD8 antibodies, indicated that CHD8 was bound to the promoters of E2F-regulated genes, such as *CCNA2*, *CDC6*, *CCNE2* and *BRCA2* (Figure 2A and Supplementary Figure S2). We thus synchronized RPE1 cells at the G0/G1 phase by serum starvation and then induced cell cycle re-entry by serum re-addition. Flow cytometry analysis demonstrated that 15% of the cells had reached S phase 14 h after serum addition, so we considered this time point to be the late-G1/S transition. Interestingly, CHD8 was almost absent from E2F-regulated promoters in quiescent cells, whereas its occupancy was increased between 5- and 20-fold by 14 h after serum restoration (Figure 2A). Levels of CHD8 mRNA were not altered by serum deprivation or serum re-addition (Supplementary Figure S3A). However, amount of CHD8 protein increased about 3-fold 14 h after serum restoration (Supplementary Figure S3B). These data suggest that both promoter binding and stability of CHD8 are proliferation-regulated processes. Next, we investigated the effect of CHD8 depletion by short interfering RNAs (siRNA) on the expression of E2F targets. As expected, *CCNA2*, *CDC6*, *CCNE2 *and *BRCA2 *genes were expressed in asynchronous cultures and at the G1/S transition (14 h time point), but their expression in quiescent cells was low. Importantly, CHD8 knockdown (Figure 2B) strongly impaired expression of these genes at the G1/S transition (Figure 2C). Similar results were obtained with three siRNA molecules that target different regions of the *CHD8* mRNA (Supplementary Figure S4A). Taken together, these data indicate that CHD8 is an essential factor in controlling gene expression at the G1/S transition.

### CHD8 is necessary for cell cycle re-entry.

We next decided to investigate the effect of CHD8 depletion on the kinetics of cell cycle progression after serum stimulation of G0-arrested RPE1 cells. CHD8 depletion provoked a strong delay of cell cycle progression: although 88.8% of the control cells were in S or G2/M phases 20 h after serum addition, only 17.7% of the CHD8-depleted cells had started DNA replication (Figure 2D and Supplementary Table S4). Serum-starved RPE1 cells generate a microtubule-based organelle called the primary cilium (26,29). These hair-like organelles are displayed on G0 phase and are resorbed as the cells re-enter the cell cycle. Thus, as a second way to evaluate the role of CHD8 in cell cycle re-entry, we assayed the kinetic of cilia resorption. Serum restoration triggers a biphasic kinetics of ciliary disassembly, which peaks at 2 h and 24 h after serum treatment. The first wave of cilia shortening occurs at early G1-phase preceding S phase entry, whereas the second wave occurs as cells are preparing to enter the G2/M phase (26). CHD8-depleted cells showed a normal first wave of cilium disassembly 2 h after serum re-addition (Supplementary Figure S5). However, 24 h after serum re-addition, 38.4% (±5%) of the CHD8-depleted cells still presented a visible cilium, compared with 12.1% (±6%) in the control cells (Supplementary Figure S5). This indicates that the second wave of cilia disassembly, which is dependent on cell cycle progression, requires CHD8.

### CHD8 is required for promoter loading of E2F1 and E2F3

**Figure 3.**
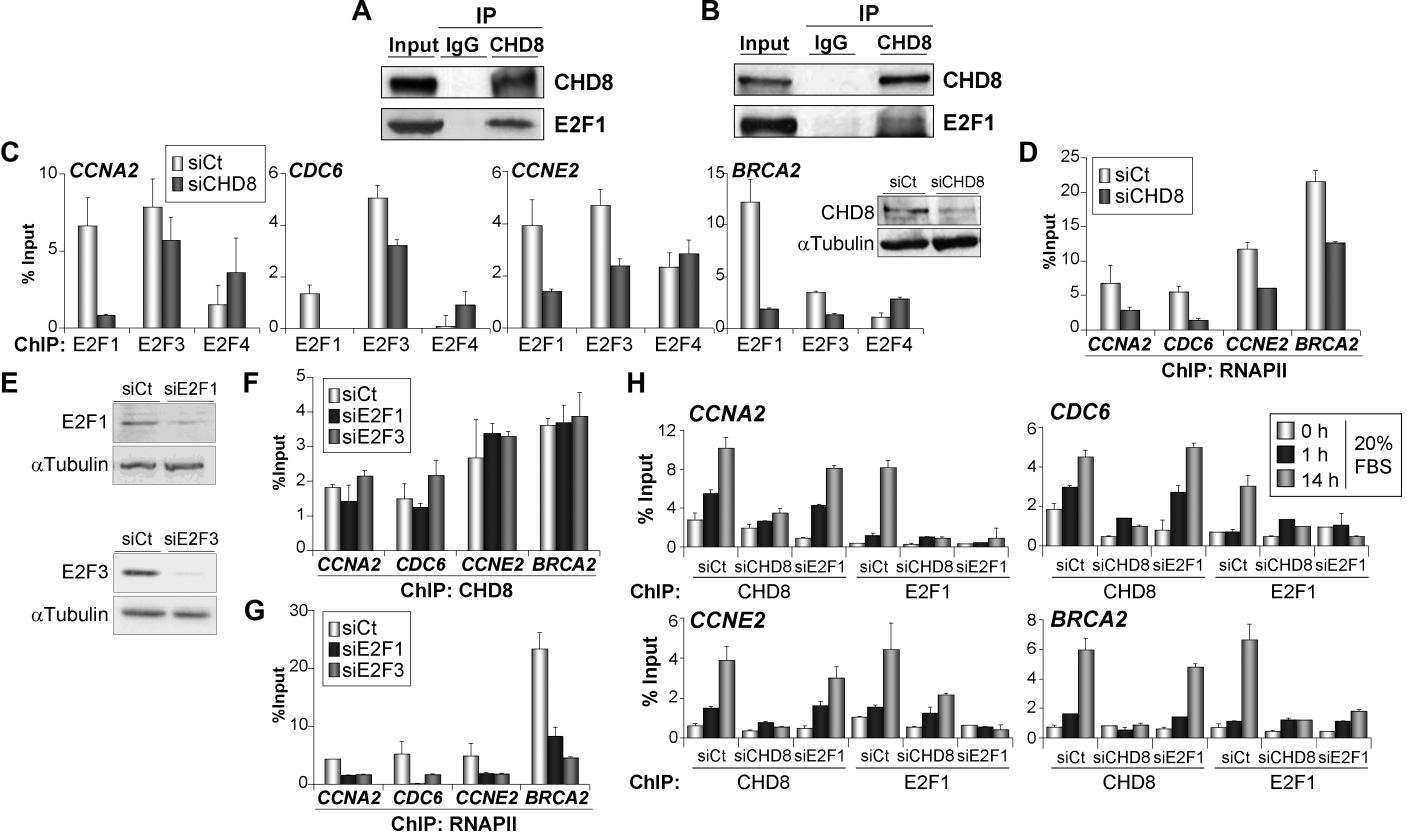
CHD8 interacts with E2F1 and it is required for E2F1 and E2F3 normal recruiting to G1/S-specific promoters. (**A** and **B**) Cell extracts from COS-7 cells transfected with a plasmid expressing E2F1 (A) or from RPE1 cells serum starved and then stimulated with 20% FBS for 14 h (B) were subjected to immunoprecipitation (IP) and analysed by western blotting with antibodies against CHD8 and E2F1. (**C** and **D**) ChIP analysis of E2F1, E2F3 and E2F4 (C) or RNAPII (D) on the indicated promoters, in exponentially growing RPE1 cells transfected with control (siCt) or CHD8-specific (siCHD8) siRNAs. Inset in (C) shows level of CHD8 expression analysed by western blotting. (**E**) E2F1 and E2F3 expression of proliferating RPE1 cells after transfection with control (siCt) or either E2F1 (siE2F1)- or E2F3 (siE2F3)-specific siRNAs, was analysed by western blotting. (**F** and **G**) ChIP analysis of CHD8 (F) and RNAPII (G) on the indicated promoters, in exponentially growing RPE1 cells transfected with control (siCt) or either E2F1 (siE2F1) or E2F3 (siE2F3)-specific siRNAs. (**H**) ChIP analysis of CHD8 and E2F1 in RPE1 cells serum starved (0 h) and then stimulated with 20% FBS for 1 or 14 h. Cells were transfected with control siRNA (siCt) or siRNA against CHD8 (siCHD8) or E2F1 (siE2F1). (C, D and F–H) Data (% input) are the mean of at least *n* = 6 qPCR reactions from three independent experiments. Error bars represent ± SD values.

The results presented earlier in text indicate that CHD8 is essential for cell cycle progression and expression of E2F-dependent genes. Therefore, we decided to investigate in detail the relationship between CHD8 and these factors. Mass spectrometry data have shown that CHD8 copurify with HCFC1 (15,16,30), a well-known E2F-interacting factor (31). Consistently, both transiently expressed and endogenous E2F1 proteins were coimmunoprecipitated with antibodies against CHD8 (Figure 3A and B), indicating that E2F1 and CHD8 form part of the same complex. Interestingly, depletion of CHD8 in exponentially growing cells impaired E2F1 and E2F3 occupancy at the analysed promoters (Figure 3C), as well as RNAPII recruitment (Figure 3D). In contrast, occupancy of E2F4 was not affected or slightly increased in CHD8-depleted cells, consistently with the G1 arrest provoked by the CHD8 deficiency. Similar E2F1 recruitment defects were obtained by using a second siRNA against CHD8 (Supplementary Figure S4B). Depletion of E2F1 or E2F3 (Figure 3E) did not significantly affect CHD8 occupancy at any of the genes tested (Figure 3F), despite the fact that RNAPII levels were decreased (Figure 3G). These data suggest that CHD8 has an essential role in mediating chromatin loading of activating E2Fs onto the G1/S-specific genes. E2F1 knockdown experiments have demonstrated a requirement for this E2F protein in allowing cells to enter cell cycle from quiescence (32). Then, we characterized the kinetics of the recruitment for CHD8 and E2F1 at the G1/S-specific promoters during cell cycle re-entry in the absence of each other. Samples were taken under quiescent conditions or 1 and 14 h after adding serum to quiescent cells. In control cells, a high level of CHD8 and E2F1 occupancy was reached 14 h after serum addition. Strikingly, a small but significant increase, more evident in the case of CHD8, was already observed after 1 h (Figure 3H), many hours before the G1/S transition. As a control, we verified that silencing CHD8 or E2F1 strongly decreased their association with chromatin, validating the ChIP signals. Consistent with the results of asynchronous cultures, depletion of CHD8 strongly impaired E2F1 recruitment. In contrast, E2F1 depletion did not significantly affect CHD8 recruitment neither at 1 h nor at 14 h on serum re-addition, indicating that CHD8 recruitment is also independent of E2F1 under these circumstances.

**Figure 4.**
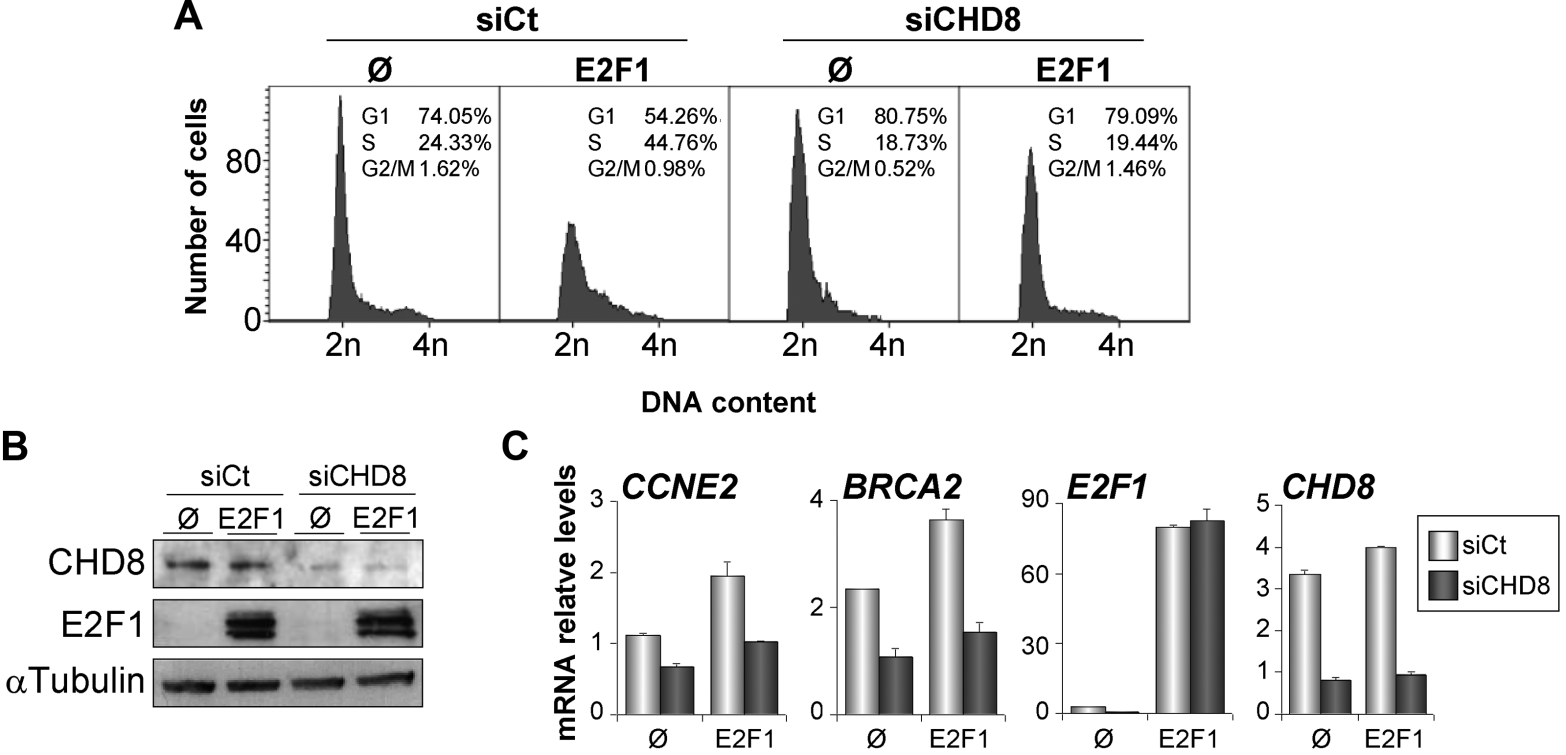
CHD8 is required for E2F1-dependent S phase stimulation of quiescent cells. (**A-C**) RPE1 cells were transfected with control (siCt) or CHD8-specific (siCHD8) siRNAs and then serum starved for 24 h. After that, cells were transfected with a plasmid expressing E2F1 (E2F1) or empty vector (Ø), maintained in serum starvation conditions for 24 h, and finally collected for cell cycle analysis by flow cytometry (A), for western blotting analysis (B) or for expression analysis by RT-qPCR (C). (B) Western blottings were performed with anti-CHD8 and anti-E2F1 antibodies. (C) Total RNA was subjected to RT-qPCR quantification with primers for the indicated genes. Data (mRNA relative level) are the mean of at least *n* = 6 qPCR reactions from three independent experiments. Error bars represent ± SD values.

Our data indicate that CHD8 is directly or indirectly required for loading activator E2F factors at G1/S transition promoters on serum stimulation of quiescent cells. To demonstrate a direct role of CHD8 in E2F function, we decided to investigate CHD8 requirement on E2F-dependent cell cycle activation in a serum-independent manner. It is well know that overexpression of E2F1 is able to trigger quiescent cells into S phase (33). Interestingly, depletion of CHD8 abolished E2F1-dependent S phase stimulation of serum-starved cells (Figure 4A and B). In agreement with the previous results, overexpression of E2F1 induced expression of the G1/S transition genes *CCNE2* and *BRCA2* in control cells but not in CHD8-depleted cells (Figure 4C), suggesting that CHD8 directly facilitates E2F1 function. Similarly, CHD8 was also required for induction of *CCNE2* and *BRCA2* genes on overexpression of the adenovirus E1A oncoprotein (Supplementary Figure S6), which it is known to overcome repression of G1/S-specific genes by disrupting the interaction of RB protein with E2Fs and other corepressors (34).

### CHD8 is required for recruitment of MLL complexes to G1/S-specific promoters

**Figure 5.**
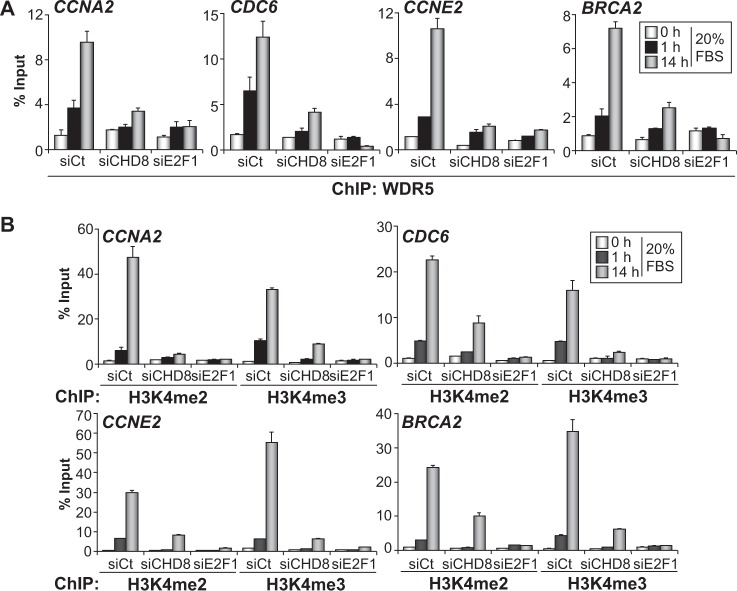
CHD8 is required for MLL complexes recruitment and histone H3K4 methylation of G1/S promoters. (**A** and **B**) ChIP analysis of WDR5 (A), H3K4me2 and H3K4me3 (B) on the indicated promoters, in RPE1 cells serum starved (0 h) and then stimulated with 20% FBS for 1 or 14 h. Cells were transfected with control siRNA (siCt) or siRNA against either CHD8 (siCHD8) or E2F1 (siE2F1). Data (% input) are the mean of at least *n* = 6 qPCR reactions from three independent experiments. Error bars represent ± SD values.

It has been reported that CHD8 copurifies with members of the MLL complexes of H3K4 methyltransferases (11,14–16). In addition, it was shown that CHD8 directly interacts with WDR5, a common component of MLL1, MLL2 and hSet1-associated complexes (14). Therefore, we also studied whether CHD8 depletion affects the recruitment of WDR5 and the methylation marks introduced by the MLL complexes. WDR5 promoter occupancy and levels of H3K4 di- and tri-methylation were determined under quiescent conditions or 1 and 14 h after serum re-addition. Control cells showed a significant augment of WDR5 occupancy and H3K4 methylation 1 h after serum addition that further increased at 14 h, indicating that MLL complexes recruitment begins many hours before the G1/S transition. We observed that depletion of E2F1 almost completely abolished recruitment of WDR5 and H3K4 di- and tri-methylation at the *CCNA2*, *CDC6*, *CCNE2 *and *BRCA2 *promoters (Figure 5A and B), indicating that other activator E2Fs play a minor role in MLL complexes recruitment to these genes, in RPE1 cells. In agreement with the fact that CHD8 is required for E2F1 loading, depletion of CHD8 also strongly reduced occupancy of WDR5 and H3K4 methylation in the four promoters analysed (Figure 5A and B). These results place CHD8 recruitment as an event required for E2F1 loading and the subsequent recruitment of MLL complexes.

## DISCUSSION

Here, we demonstrate that the chromatin remodeller CHD8 binds the promoters of a subset of transcriptionally active genes enriched in H3K4me2 and H3K4me3 marks, many of which are related to macromolecular biosynthesis and cell proliferation. Additionally, we show that CHD8 leaves G1/S gene promoters under quiescent conditions. CHD8 recruiting occurs shortly after serum stimulation of quiescent cells, far earlier than transcription is activated, suggesting that the chromatin of G1/S transition genes is starting to change several hours before the G1/S transition. Finally, we demonstrate that CHD8 is required for the recruitment of E2F1, E2F3, MLL complexes and RNAPII to G1/S transition promoters, and therefore for E2F-dependent transcription activation and cell cycle progression.

Our data now demonstrate that CHD8 plays a positive role in transcription, in addition to its previously reported in repressing p53- and β-catenin-dependent gene expression (10–13). Specifically, our ChIP-chip experiments showed that CHD8 mostly binds to promoters of transcriptionally active genes that also contain high levels of H3K4me2 and H3K4me3, two post-transcriptional modifications typical for active promoters. In addition, CHD8 was absent or poorly enriched in repressed genes. We also demonstrated that artificial recruitment of CHD8 to a minimal promoter provoked transcription activation. Furthermore, expression of G1/S transition genes was impaired in CHD8-depleted cells. Similarly to CHD8, the *Drosophila* orthologous Kismet is found in transcriptionally active genes in polytene chromosomes (35). Obviously, our data do not preclude that CHD8 also plays a role in repression. Roles as activators and repressors have been described for other remodellers of the SNF2 family and their associated complexes (36,37).

The CHD subfamily of SNF2 proteins is characterized by the presence of two tandem chromodomains that are amino terminal to the SNF2 ATPase/helicase domain. There are three distinct groups within the human CHD subfamily: CHD1/2, CHD3/4/5, and CHD6/7/8/9 (8). CHD6/7/8/9 are paralogous genes related to the *Drosophila Kismet *gene. Genome-wide studies have shown that CHD7 is mostly associated to a subset of active enhancers and promoters in mice ES cells (38). Furthermore, it has been recently shown that CHD7 interacts with CHD8 (39). Using ENCODE ChIP-seq data of the distribution of CHD7 in K562 cells, we have found that 5.7% (112) of the CHD8-bound promoters in C33A cells are bound by CHD7. Among the overlapping genes, those for ribosomal proteins (*P* = 5.7 × 10^−^^9^) and chromatin architectural proteins (*P* = 4.4 × 10^−^^13^) are highly represented. The low number of overlapping genes may be the consequence of the different cell lines used for the study. Nevertheless, gene ontology analysis of the CHD7-bound genes did not indicate any association with cell cycle genes, suggesting that although CHD8 and CHD7 may share a number of targets, they also have gene-specific targets.

We have previously shown that CHD8 depletion in asynchronous cultures provokes a growth defect and a small G1 arrest in the C33A tumour cell line (17). In this cell line, CHD8 was constitutively bound to the promoter of *CCNE2* gene throughout the cell cycle, as well as in serum-deprived cells [(17) and our unpublished results]. However, we failed to achieve growth arrest by serum deprivation in C33A cells, and therefore decided to use the non-transformed cell line of RPE1. With these cells, we showed that CHD8 occupancy at G1/S target gene promoters dramatically decreased in quiescent cells, and that its binding to G1/S promoters, including *CCNE2*, was dependent on serum re-addition. Furthermore, we showed that CHD8 was absolutely required for cell cycle progression following serum re-addition. As growth factors-independent proliferation is a hallmark of many types of tumours (40), it will be interesting to investigate whether the presence of CHD8 in the absence of serum at G1/S promoters is a general characteristic of cancer cells.

**Figure 6.**
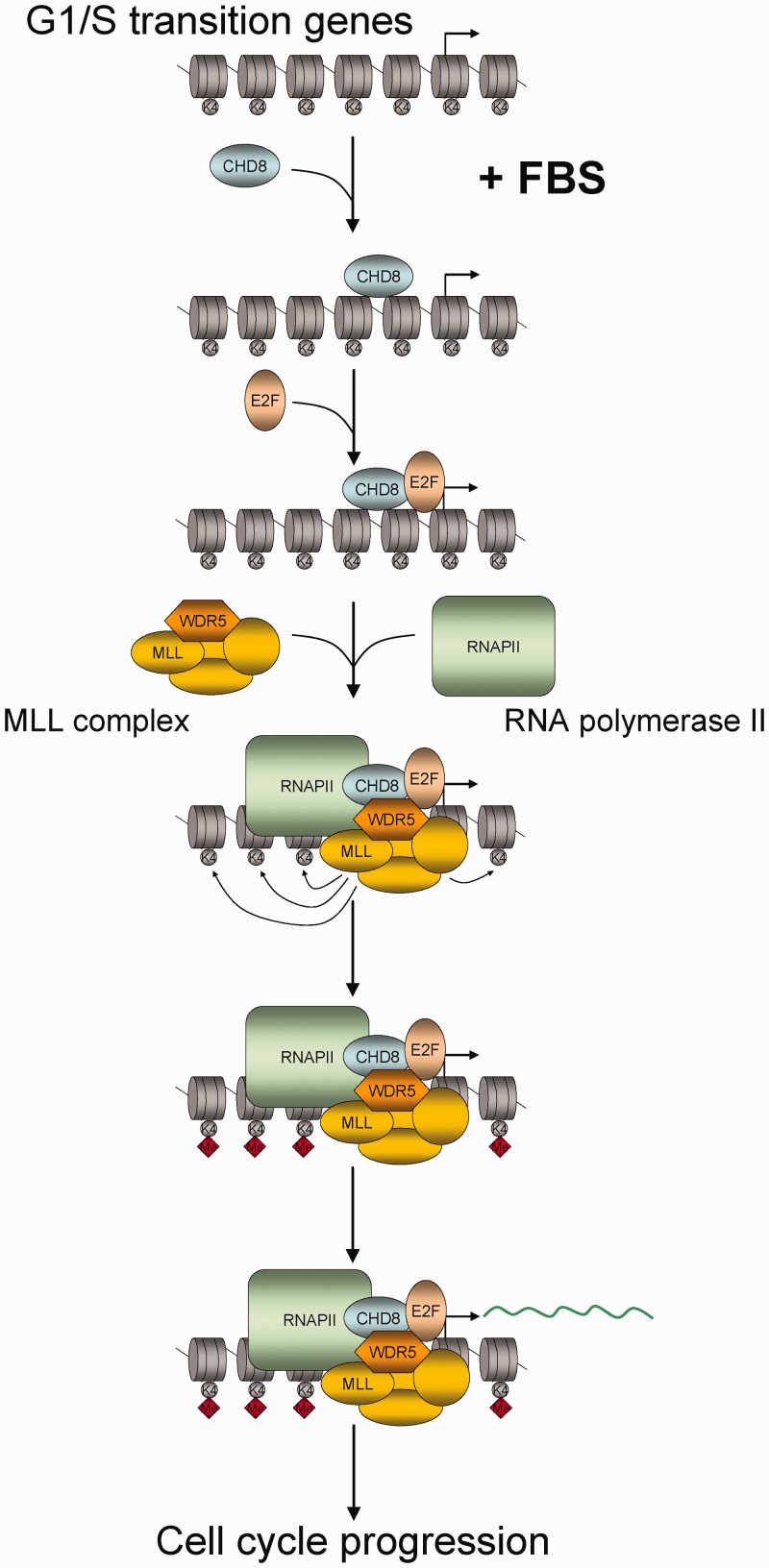
Schematic diagram indicating the proposed order of events occurring at E2F target promoters on serum activation of quiescent cells.

We show that CHD8 binds to more than a thousand promoters containing E2F binding sites, including many G1/S transition-specific genes. E2F-dependent transcription activation is a finely controlled process that requires extensive chromatin changes from a repressed RB-mediated state, characterized by the presence of repressive histone marks such as H3K9m2/3, H3K27me2/3, H4K20me and low acetylation, to an active state characterized by acetylated histones and methylated H3K4 (7). This is an interesting example of how Polycomb complexes and Polycomb-mediated histone marks are substituted by TrxG complexes and marks in a not yet fully understood way (41,42). We demonstrate that depletion of CHD8 (a human TrxG protein) severely impaired E2F1 and E2F3 loading onto 4 different E2F-dependent promoters, suggesting that CHD8 is required for the substitution of the repressor E2F factors by the activator E2Fs. Furthermore, depletion of CHD8 also impairs WDR5 occupancy and H3K4 methylation at these promoters. The WDR5/Ash2L/RbBP5 subcomplex forms part of several MLL complexes but can also form a subcomplex independently of MLL proteins (43,44). It has been shown that CHD8 directly interacts with the three components of the WDR5/Ash2L/RbBP5 subcomplex (14). However, our data demonstrate that in the absence of E2F1, CHD8 binds but WDR5 does not bind to G1/S promoters, suggesting that CHD8 is loaded onto the chromatin independently of the WDR5/Ash2L/RbBP5 subcomplex and before H3K4 methylation occurs. How does CHD8 activate transcription is by the moment unclear. CHD8, through its ATP-dependent chromatin remodelling activity, may contribute to promote an open chromatin configuration that allows activator E2Fs binding. We have shown that the ATPase activity of CHD8 is partially required for transcription activation of a chimeric promoter, indicating that part of its transactivation activity is independent of the remodelling activity. It has been previously shown that CHD8 interacts with RNAPII (17) and with subunits of the MLL complexes (11,14–16), and in this work we show that CHD8 also interacts with E2F1. Taken together, these data suggest that CHD8 also acts as a scaffolding protein that establishes contacts with several components essential for the activation of G1/S promoters, and that its absence avoids subsequent steps of gene activation. Therefore, our results suggest an order to the events occurring at E2F target promoters on activation of quiescent cells, where CHD8 binding and activity precedes activators E2F binding, that then lead to MLL complexes binding, H3K4 methylation and RNAPII recruitment (Figure 6). However, one issue remains obscure: how is CHD8 recruited to chromatin? Analysis of the more common transcription factor binding sites in the CHD8-bound promoters showed that, in addition to E2F sites, ELK1 binding sites were also strongly represented (9.19 × 10^−^^39^). ELK1 is directly activated through its phosphorylation by MAPK on growth factors stimulation (45). ELK1 can function in transcriptional activation by cooperating with the serum response factor (SRF) through protein–protein interactions and also through the close positioning of their DNA binding sites (46,47). Interestingly, an interaction of CHD8 with serum response factor has been previously reported (48). Another possibility is that CHD8 is recruited by its interaction with a chromatin mark. We have previously shown that the chromodomains of CHD8 interact with H3K4 methylated peptides *in vitro*, with a strong preference for H3K4me2 (17). Consistently with these results, now we observe that the profile of CHD8 occupancy around TSS exactly matches that of H3K4me2 (Figure 1B), suggesting that CHD8 also displays higher affinity for this modification *in vivo*. We also show that H3K4 methylation was dependent on CHD8 in G1/S promoters, and therefore it seems unlikely that H3K4 methylation is essential for CHD8 recruitment. However, we cannot exclude that CHD8 activity triggers a positive feed-forward loop for activator complexes assembly where binding of activator E2F and MLL complexes increases H3K4 methylation and promotes stabilization and additional recruitment of CHD8 at the promoters.

Another ATP-dependent chromatin remodelling machine also play a role in E2F-dependent activation: a subset of SWI/SNF complexes (also called BAF complexes) containing the ARID1B subunit are associated with E2F-dependent genes at late G1 and S phases and depletion of ARID1B impairs expression of E2F targets (49). What is the interplay between SWI/SNF and CHD8 and what are the specific events of nucleosome remodelling performed by each machinery are unsolved questions. However, it is worth noting that CHD7, a CHD8 paralogue, interacts with SWI/SNF complexes (50). Furthermore, peptides from CHD8 were discovered in a proteomic analysis of SWI/SNF complexes, so the two machineries may functionally interact (51).

All together, our data reveal an essential role for CHD8 in cell cycle re-entry and progression. Alterations in the regulation of the RB-E2F pathway are common events in cancer (3). Thus, inactivation of the tumour suppressor gene RB or deregulated expression of E2F proteins has been detected in many human cancers. Here we show that depletion of CHD8 was able to suppress E2F1 overexpression-mediated induction of cell cycle in quiescent cells. Furthermore, analyses of publicly available gene expression sets using the ONCOMINE or NEXBIO databases revealed that CHD8 is significantly upregulated in esophageal adenocarcinoma (*P* = 6.32 × 10^−^^5^) (52), ovarian carcinoma (*P* = 1.98 × 10^−^^9^) (53), lung adenocarcinoma (*P* = 2.9 × 10^−^^9^) (54) and prostate cancer (*P* = 0.0009) (55), suggesting a positive role of CHD8 in tumour formation. We believe that these results support investigating a possible role for CHD8 inhibitors as anti-cancer drugs.

## SUPPLEMENTARY DATA

Supplementary Data are available at NAR Online, including [56,57].

## FUNDING

Spanish Ministerio de Ciencia e Innovacion [BFU2011-23442, CSD2006-00049 and an FPU fellowship to E.V.C.]; Junta de Andalucía [P06-CVI-4844]; and Fundación Ramón Areces. JAE grant from C.S.I.C (to A.S.R.). Funding for open access charge: Spanish Ministerio de Ciencia e Innovacion [BFU2011-23442].

*Conflict of interest statement*. None declared.

## Supplementary Material

Supplementary Data
