# Supplementary material for: The chromatin remodeller CHD8 is required for E2F-dependent transcription activation of S-phase genes
Source: Nucleic Acids Res. 2013 Nov 20;42(4):2185–96. doi: 10.1093/nar/gkt1161 (PMC3936757; doi:10.1093/nar/gkt1161)
Supplement: Supplementary Data [file supp_42_4_2185__index.html]

The chromatin remodeller CHD8 is required for E2F-dependent transcription activation of S-phase genes — The chromatin remodeller CHD8 is required for E2F-dependent transcription activation of S-phase genes — Supplementary Data 

# The chromatin remodeller CHD8 is required for E2F-dependent transcription activation of S-phase genes

## Supplementary Data

files

**Files in this Data Supplement:**

- Supplementary Data - pdf file
- Supplementary Data - pdf file
- Supplementary Data - pdf file
- Supplementary Data - xls file
- Supplementary Data - xls file
