# Supplementary material for: The chromatin remodeller CHD8 is required for E2F-dependent transcription activation of S-phase genes
Source: Nucleic Acids Res. 2013 Nov 20;42(4):2185–96. doi: 10.1093/nar/gkt1161 (PMC3936757; doi:10.1093/nar/gkt1161)
Supplement: Supplementary Data [file supp_gkt1161_nar-02071-m-2013-File008.pdf]

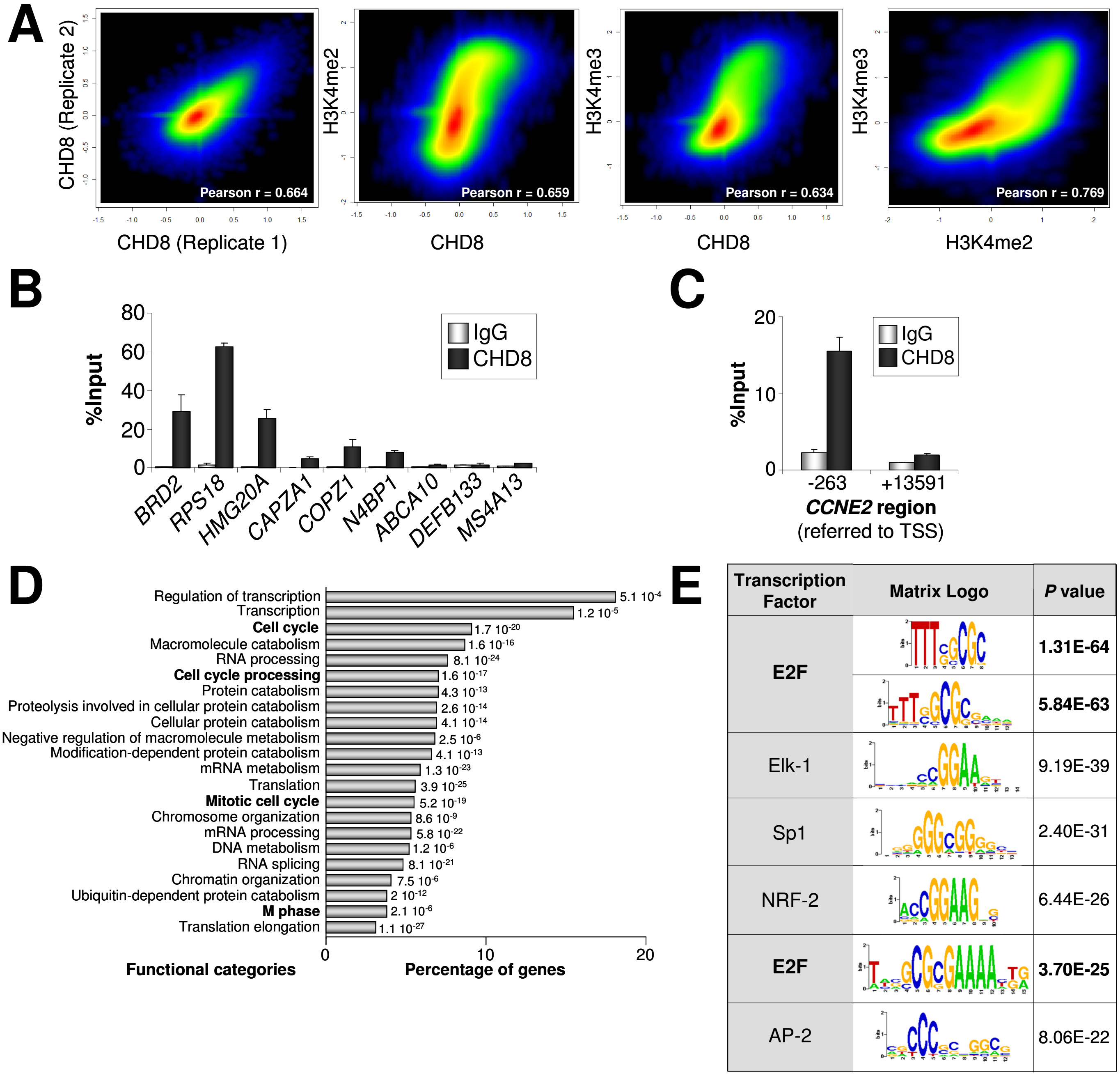

Figure S1. Genome wide analysis of CHD8 targets by ChIP-chip.

(A) Scatter plot of log-ratios/input for CHD8 binding between the two replicates of CHD8 ChIP-chip experiments, or the enrichment of CHD8 compared to H3K4me2, CHD8 compared to H3K4me3 or H3K4me2 compared to H3K4me3. Each point corresponds to a single probe. **(B-C)** C33A cells were grown exponentially and then collected for ChIP experiments with anti-CHD8 antibody or rabbit IgG as a control. Precipitated DNA fragments were subjected to qPCR quantification with primers for the indicated genes. Data (% input) are the mean of at least n = 6 qPCR reactions from three independent experiments. Error bars represent ± SD values. **(D)** Gene ontology functional categories of CHD8 target genes, analysed by DAVID (28). The numbers at the horizontal axis are the percentage of CHD8 target genes within a functional category. The numbers at the right of the bars represent the *P* value for the statistical over-representation in each category. Categories highlighted in bold are related to cell cycle. **(E)** The top transcription factor binding motifs in CHD8 binding genes, analysed using TRANSFAC database (56) and Weeder PScan (57). The associated logo with the matrix and the statistical enrichment (*P* value) are also shown.

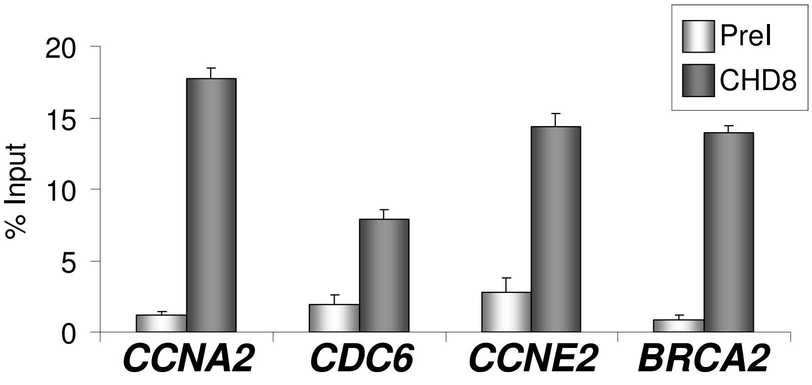

Figure S2. CHD8 binds to E2F-dependent genes.
ChIP analysis of CHD8, using a home-made anti-CHD8 antibody (17), on selected E2Fdependent genes, in exponentially growing RPE1 cells. Data (% input) are the mean ofat least n = 6 qPCR reactions from three independent experiments. Error bars represent ± SD values. PreI, pre-immune serum.

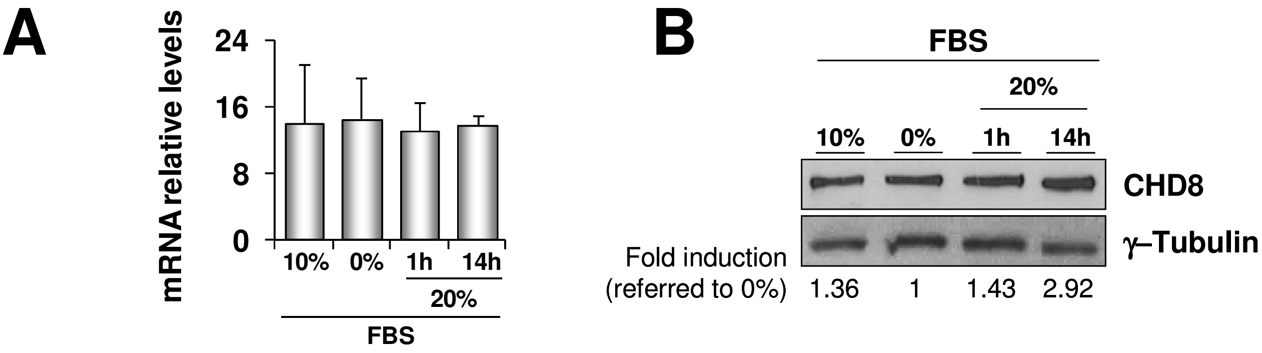

Figure S3. CHD8 expression in exponentially growing cells or upon serum readdition to quiescent cells.

RPE1 cells exponentially grown (10%) or serum-starved for 48 h (0%) and then serum stimulated (FBS 20%) for the indicated times were collected for expression analysis. **(A)** Level of *CHD8* mRNA was determined by RT-qPCR with specific primers. Data (mRNA relative level) are the mean of at least n = 6 qPCR reactions from three independent experiments. Error bars represent ± SD values. **(B)** Protein level of CHD8 was determined by Western blotting with anti-CHD8 antibody. Western blotting with anti-γ-tubulin antibody was used as a loading control. The numbers below corresponds to the quantification by ImageJ Densitometry Software.

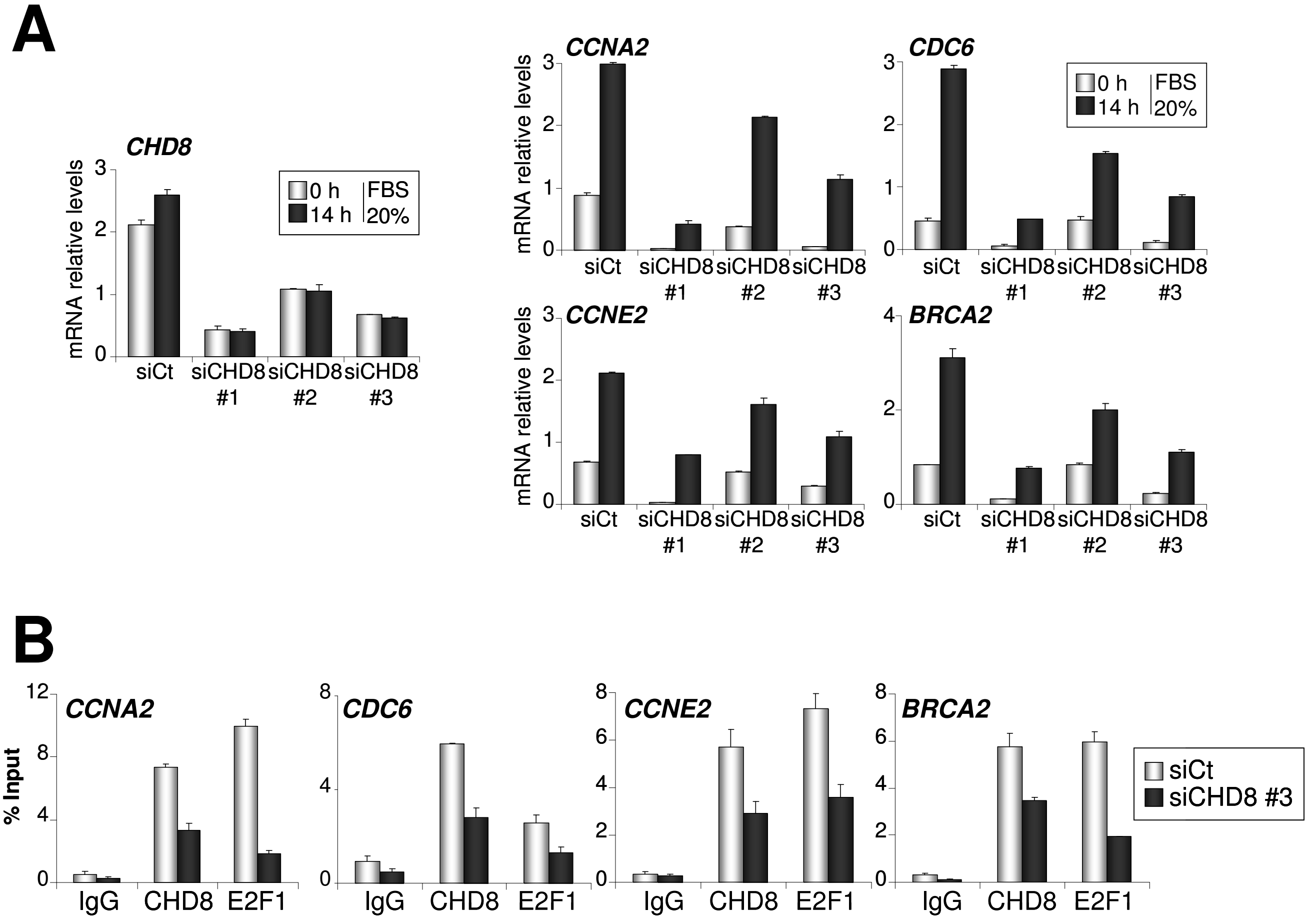

Figure S4. CHD8 controls expression and is required for E2F1 recruiting to G1/S specific promoters.

(A) RT-qPCR analysis of expression of selected G1/S specific genes in RPE1 cells transfected with control siRNA or three independent siRNA against CHD8 (siCHD8 #1, siCHD8 #2 and siCHD8 #3). Cells were serum starved for 48 h (0h) and then stimulated with 20% FBS for 14 h (14h). Efficiency of CHD8 knockdown was analyzed by qPCR with primers for *CHD8* gene. **(B)** ChIP analysis of CHD8 and E2F1, in exponentially growing RPE1 cells transfected with control siRNA (siCt) or an alternative siRNA against CHD8 (siCHD8 #3). **(A, B)** Data (mRNA relative level or % input) are the mean of at least n = 6 qPCR reactions from three independent experiments. Error bars represent ± SD values.

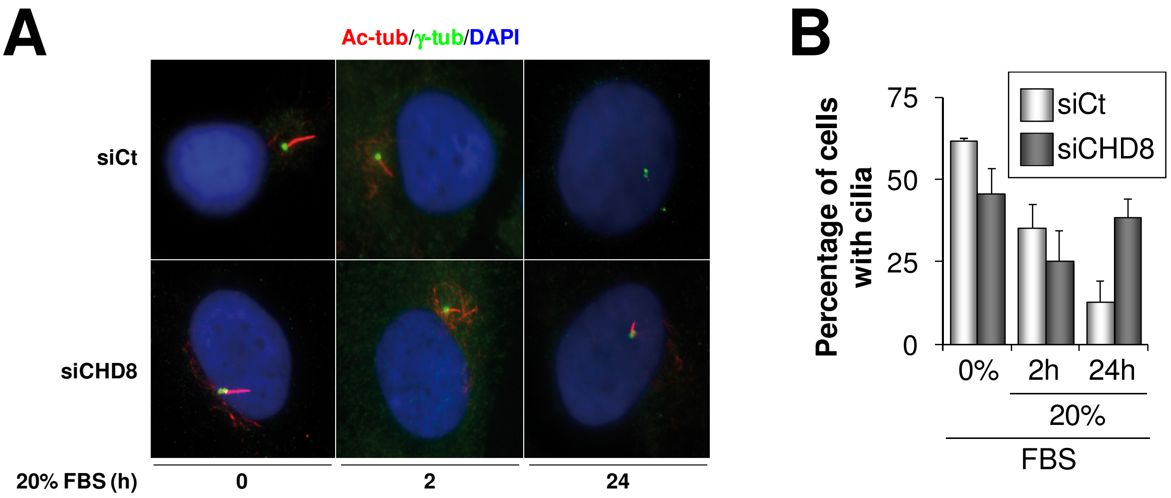

Figure S5. CHD8 is essential for cilium resorption upon serum re-addition to quiescent cells.

(A) Control (siCt) or CHD8-depleted (siCHD8) RPE1 cells were serum starved for 48 h to promote cilia assembly. Cells were then serum stimulated with 20% FBS for 0, 2 or 24 h, fixed and subjected to immunofluorescence microscopy with antibodies against acetylated α-tubulin (Ac-tub; red) and γ-tubulin (γ-tub; green). DAPI was also used to stain nuclear DNA (blue). A representative experiment is shown. **(B)** Quantification of fraction of cells with cilium. Cells were treated and subjected to immunofluorescence as indicated in (A). An average of 150 cells was counted from three independent experiments. Error bars represent ± SD values.

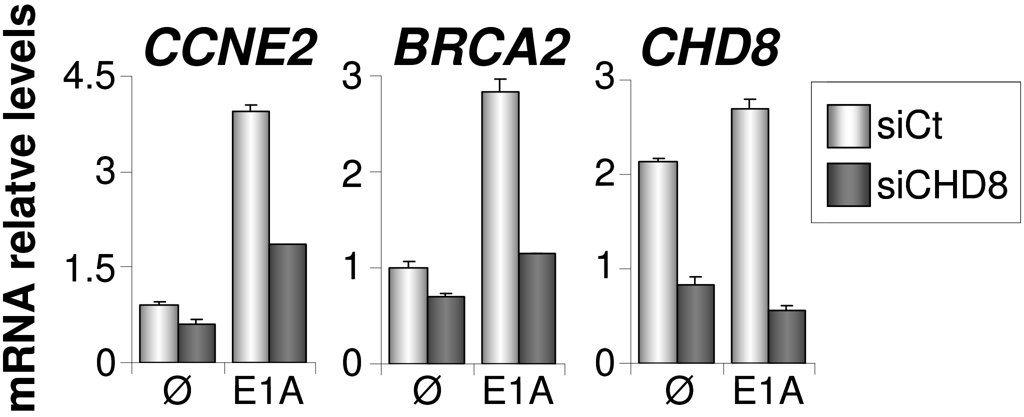

Figure S6. CHD8 is required for E1A-dependent S phase genes expression in quiescent cells.

RPE1 cells were transfected with control siRNA (siCt) or siRNA against CHD8 (siCHD8) and then serum starved for 24 h. After that, cells were transfected with a plasmid expressing E1A (E1A) or empty vector (Ø), maintained in serum starvation conditions for 24 h, and finally collected for expression analysis by RT-qPCR. The obtained cDNAs were subjected to qPCR quantification with primers for the indicated genes. Data (mRNA relative level) are the mean of at least n = 6 qPCR reactions from three independent experiments. Error bars represent ± SD values.
