# Supplementary material for: The chromatin remodeller CHD8 is required for E2F-dependent transcription activation of S-phase genes
Source: Nucleic Acids Res. 2013 Nov 20;42(4):2185–96. doi: 10.1093/nar/gkt1161 (PMC3936757; doi:10.1093/nar/gkt1161)
Supplement: Supplementary Data [file supp_gkt1161_nar-02071-m-2013-File009.pdf]

Oligos for ChIP		
**Gene**	**Sequence**	**Position (referred to TSS)**
ABCA10	5'-TTTTAACAAACTCAGGGCACAA-3'	+37
	5'-ACTAACAACGTTTTATAGGAGCACAA-3'	
BRCA2	5'-AAGCGTGAGGGGACAGATT-3'	+135
	5'-GCCGGAGTAAGCTGACAAA-3'	
BRD2	5'-GCAGTAACCGAGGTTGGAGT-3'	-427
	5'-GGCCTAGGTCCTTGAGCTG-3'	
CAPZA1	5'-TTACCGCTTCCAAACGAAAT-3'	-14
	5'-TTTTTCTGGTGCCCGTTATC-3'	
CCNA2	5'-CAGTGCCTGGGGTTTAAAAG-3'	+805
	5'-GGGGTCAAACCAAGCTCTAA-3'	
CCNE2	5'-TGACACCCCCGAAATCCA -3'	-263
	5'-CCTGGCTCGCGCATCT-3'	
	5'-GGAATTTCGTTCCTCCCTCT-3'	+1235
	5'-CAGGCCAAGAAGAGGAAAACT-3'	
	5'-CAGTGTGCAATGGAGGCATT-3'	+13591
	5'-AGTGTTTTCCTGGTGGTTTTTCA-3'	
CDC6	5'-GTAAAAGCCCTGCCTCTCAG-3'	+1694
	5'-ATGGATGGTTTACCCAGACG-3'	
COPZ1	5'-GGCATAGAGTCGGTGGAAAG-3'	+338
	5'-GCAACCTGAAGGGTCCTAATC-3'	
DEFB133	5'-CTCCAGTTGACATCGACCAA-3'	+80
	5'-TTTCTTTCTGGTCCCCATTG-3'	
HMG20A	5'-CCACGAAAATAAGGCTCACC-3'	-182
	5'-ATTGCTCCGGTGAGAAGAAA-3'	
MS4A13	5'-GAGGTTTGCAGAAACCGTTC-3'	-140
	5'-GTGTGCGCAGACTCACTAGG-3'	
N4BP1	5'-AGGAGGCGAGACTGAGAGTG-3'	+290
	5'-TCTGCCTGTGGATTAACGTG-3'	
RPS18	5'-TCCCCTTTCAGCTCTAACCA-3'	-189
	5'-GCTTGGGTACCACAACTTCC-3'	

Oligos for RT-PCR	
**Gene**	**Sequence**
ACTB	5'-ACGAGGCCCAGAGCAAGA-3'
	5'-GACGATGCCGTGCTCGAT-3'
BRCA2	5'-AGGCTTCAAAAAGCACTCCA-3'
	5'-TTGTGCGAAAGGGTACACAG-3'
CCNA2	5'-TTATTGCTGGAGCTGCCTTT-3'
	5'-CTCTGGTGGGTTGAGGAGAG-3'
CCNE2	5'-TTGGCTATGCTGGAGGAAGT-3'
	5'-TGACAACTGTCCCCCTTTTC-3'
CDC6	5'-CTTAAGCCGGATTCTGCAAG-3'
	5'-CAGTCCTCAAGGACATGCAA-3'
CDKN1B	5'-TGCAACCGACGATTCTTCTA-3'
	5'-CGAGCTGTTTACGTTTGACG-3'
CHD8	5'-TTGAGCAAGCTGTCACCATC-3'
	5'-GAGCGGGACTGGTCAGTTAG-3'
E2F1	5'-AGCTGGACCACCTGATGAAT-3'
	5'-GAGGGGCTTTGATCACCATA-3'
GAPDH	5'-GAGTCAACGGATTTGGTCGT-3'
	5'-AATGAAGGGGTCATTGATGG-3'

Table S1
