# Supplementary material for: The chromatin remodeller CHD8 is required for E2F-dependent transcription activation of S-phase genes
Source: Nucleic Acids Res. 2013 Nov 20;42(4):2185–96. doi: 10.1093/nar/gkt1161 (PMC3936757; doi:10.1093/nar/gkt1161)
Supplement: Supplementary Data [file supp_gkt1161_nar-02071-m-2013-File012.pdf]

			G1	S	G2-M
	10%	siCt	**46.57** ± 10.01	**37.33** ± 9.92	**16.10** ± 1.17
		siCHD8	**83.07** ± 5.76	**11.90** ± 6.58	**4.60** ± 0.22
20% FBS	0h	siCt	**96.17** ± 2.16	**2.91** ± 1.73	**1.00** ± 0.69
		siCHD8	**96.40** ± 1.61	**2.25** ± 0.35	**1.34** ± 1.53
	1h	siCt	**93.96** ± 2.27	**2.78** ± 0.97	**2.12** ± 0.42
		siCHD8	**95.28** ± 0.35	**2.31** ± 0.96	**2.41** ± 1.54
	12h	siCt	**90.30** ± 0.94	**7.35** ± 0.76	**2.36** ± 0.18
		siCHD8	**95.65** ± 0.35	**2.14** ± 0.96	**2.21** ± 0.62
	14h	siCt	**82.06** ± 1.78	**16.74** ± 3.12	**1.20** ± 1.34
		siCHD8	**95.67** ± 0.09	**2.17** ± 1.12	**2.17** ± 1.20
	16h	siCt	**62.38** ± 7.69	**37.63** ± 7.69	**2.13** ± 0.69
		siCHD8	**96.52** ± 0.47	**2.62** ± 0.01	**0.87** ± 0.46
	20h	siCt	**11.15** ± 3.02	**61.11** ± 9.76	**27.74** ± 6.65
		siCHD8	**82.34** ± 2.30	**17.04** ± 3.14	**0.63** ± 0.83
	24h	siCt	**18.80** ± 3.17	**24.05** ± 3.77	**57.16** ± 6.94
		siCHD8	**55.07** ± 8.49	**41.13** ± 7.28	**4.30** ± 0.51
	36h	siCt	**46.19** ± 8.97	**45.24** ± 9.81	**8.57** ± 4.55
		siCHD8	**57.99** ± 9.29	**15.05** ± 3.49	**26.96** ± 6.49
	48h	siCt	**61.53** ± 9.85	**27.34** ± 7.25	**11.13** ± 3.22
		siCHD8	**57.61** ± 8.53	**19.16** ± 4.15	**23.23** ± 5.12

Table S4. Quantification of cell cycle phases from Figure 2D.
Numbers are average percentages from three independent experiments.
